# Child growth trajectories to adult disease: lessons from UK birth cohort studies

**DOI:** 10.1186/1687-9856-2015-S1-O1

**Published:** 2015-04-28

**Authors:** Ken K Ong

**Affiliations:** 1 MRC Epidemiology Unit, Institute of Metabolic Science, University of Cambridge, Cambridge, UK

## 

The developmental origins of health and disease theory purports that early life factors determine long-term risks of death and disease. Historical studies, prospective birth cohorts such the UK ALSPAC birth cohort [1], and more recently genetic studies [2] indicate that the rapid weight gain trajectory to later obesity starts in the first months of life, even from birth. Rapid infant weight gain and childhood overweight lead to earlier pubertal maturation in boys and girls, and in turn these adolescent traits are predictive for obesity, diabetes, hypertension and cardiovascular disease events in later life. Understanding of the nutritional, parental and wider determinants of rapid infant weight gain are informing the development of obesity prevention strategies starting in early life [3].

In contrast to the above ‘rapid growth tempo’ trajectory to later disease, poor childhood growth is also a risk factor for later health and survival. This ‘early childhood stunting’ trajectory is seen in older UK birth cohorts, such as the 1946 British Birth Cohort Study [4], and is likely relevant to current children in lower and middle-income countries. The relative importance of these distinct childhood trajectories for later health likely depends on the prevailing nutritional environment. However, in those populations that are undergoing rapid nutritional transition from under- to over-nutrition, the adverse combination of early childhood stunting followed by transition to overweight and obesity will be particularly detrimental to later health.
